# High-frequency ultrasound features of cutaneous hidrocystoma: a case series with imaging-pathology correlation

**DOI:** 10.3389/fmed.2026.1849981

**Published:** 2026-06-26

**Authors:** Lina Kong, Xiao Tang, Kunming Pu, Xiachuan Qin

**Affiliations:** Department of Ultrasound, West China School of Medicine, Sichuan University, Sichuan University Affiliated Chengdu Second People's Hospital, Chengdu Second People's Hospital, Chengdu, Sichuan, China

**Keywords:** diagnosis, hidrocystoma, histopathology, preoperative evaluation, ultrasound

## Abstract

**Background:**

Cutaneous hidrocystoma is an uncommon benign cystic lesion of sweat gland origin that frequently presents diagnostic challenges. While clinical examination remains the first-line diagnostic approach, with dermoscopy increasingly used in dermatology practice, high-frequency ultrasound (HFUS) may provide additional valuable information for lesion characterization and surgical planning. However, systematic characterization of HFUS features in hidrocystoma remains limited. This study aimed to describe the sonographic features of pathologically confirmed cutaneous hidrocystoma, correlate these findings with histopathologic features, and summarize the potential role of HFUS in preoperative lesion localization and differential diagnostic assessment.

**Methods:**

Eleven patients with pathologically confirmed cutaneous hidrocystoma were analysed. All patients underwent clinical examination by dermatologists prior to ultrasound evaluation. Ultrasound examinations were performed by two board-certified radiologists with more than 10 years of experience in superficial soft-tissue imaging, using high-frequency linear transducers. Lesion location, size, morphology, margin, internal echogenicity, vascularity, and relationship to adjacent structures were systematically evaluated and correlated with histopathologic findings. Preoperative ultrasound findings were compared with postoperative histopathologic results.

**Results:**

Eleven lesions were identified in 11 patients (6 men and 5 women; age range, 27–71 years; mean age, 48.0 years). Eight lesions were located in the head and neck region. Most lesions measured less than 1.5 cm (10/11, 90.9%). All lesions were located within the dermis or superficial subcutaneous layer. Sonographic appearances were variable: heterogeneous echogenicity (36.4%), anechoic cystic features (27.3%), homogeneously hypoechoic (18.2%), and hypoechoic with papillary projections (18.2%). Blood flow signals were detected in 2 lesions (18.2%): one exhibited minimal vascularity, whereas the other demonstrated prominent peripheral vascularity with a circumferential encasing pattern. Preoperative ultrasound interpretations included cystic lesion (3 cases), epidermoid cyst (4 cases), hemangioma (2 cases), and hypoechoic nodule (2 cases). None of the lesions was interpreted as hidrocystoma on preoperative ultrasound.

**Conclusion:**

Although HFUS features of cutaneous hidrocystoma show certain patterns, they overlap with atypical epidermoid cysts and hemangiomas, limiting definitive preoperative diagnosis. Nevertheless, HFUS allows precise delineation of lesion depth, internal architecture, and vascular relationships, thereby facilitating accurate localization, narrowing the differential diagnosis, and supporting individualized surgical planning.

## 1 Introduction

Cutaneous hidrocystoma is an uncommon benign cystic lesion of sweat gland origin (1). Its pathogenesis has not been fully elucidated, although ductal obstruction, functional abnormalities of the sweat glands, and, in some cases, genetic or syndromic associations have been proposed as contributing factors (1). Clinically, hidrocystoma occurs most often in middle-aged and older adults and shows a predilection for the head and neck region, particularly the periocular area (1, 2). It usually presents as a slowly growing, asymptomatic, smooth, dome-shaped cystic papule or nodule measuring only a few millimeters to approximately 1.5 cm in diameter, with a translucent, bluish, or blue-black appearance (1–3). Because the clinical presentation is often nonspecific, cutaneous hidrocystoma may be mistaken for other superficial lesions, including epidermoid cysts, vascular lesions, and peripheral nerve sheath tumors, thereby creating challenges for preoperative diagnosis (1, 4, 5).

In routine clinical practice, diagnosis of cutaneous hidrocystoma typically begins with clinical examination by dermatologists, with dermoscopy increasingly employed as a non-invasive adjunctive tool to evaluate surface features and vascular patterns (6). However, dermoscopy has limitations in assessing lesion depth, internal architecture, and relationship to adjacent structures. High-frequency ultrasound (HFUS) has emerged as a complementary imaging modality that addresses these limitations.

It should be noted that the terminology for high-frequency ultrasound in dermatology requires clarification. According to the dermatologic ultrasound literature, high-frequency ultrasound (HFUS) typically refers to frequencies of 15 MHz or greater, with 20–30 MHz being the most commonly used range for detailed skin layer evaluation (7, 8). The minimum frequency for dermatological analysis is considered to be 15 MHz (7). Ultra-high-frequency ultrasound (UHFUS) is defined as frequencies above 30 MHz, typically 48–70 MHz, which provide enhanced resolution for epidermal and superficial dermal structures (7, 8). Ultrasound using high-frequency transducers of approximately 5–15 MHz is adequate for evaluating superficial soft-tissue lesions up to approximately 2–3 cm in depth and is commonly used for the initial assessment of cutaneous and subcutaneous pathology in clinical settings where specialized dermatologic ultrasound equipment (≥ 15 MHz) is not routinely available (7, 8).

Histopathologic examination remains the reference standard for definitive diagnosis. However, its invasive nature limits its utility as a routine preoperative assessment tool. In this context, a reliable non-invasive imaging method is clinically desirable. Conventional high-frequency ultrasound (5–15 MHz) has become an important modality for the evaluation of superficial soft-tissue and cutaneous lesions because it is non-invasive, readily accessible, capable of real-time dynamic assessment, and able to provide high spatial resolution for superficial structures (6–10). In addition to identifying whether a lesion is located within the skin or superficial subcutaneous tissue, conventional HFUS can depict internal architecture, vascularity, and the relationship of the lesion to adjacent structures, all of which are relevant to differential diagnosis and surgical planning (7, 9–11).

Recently, non-invasive diagnostic imaging techniques including ultra-high-frequency ultrasound (UHFUS, ≥ 48 MHz) have gained increasing attention for the evaluation of hidrocystoma (6). Cinotti et al. (2024) demonstrated that UHFUS enables precise visualization of hidrocystoma architecture, revealing anechoic or hypoechoic oval structures with thin hyperechoic borders and internal septa in the majority of cases (6). Despite these advantages, systematic sonographic studies of cutaneous hidrocystoma remain limited, and its ultrasonographic features have not been sufficiently summarized. Existing reports are largely confined to case reports or lesions in specialized anatomic sites, such as the eyelid or periocular region (2, 6, 12, 13).

Accordingly, the present study retrospectively analysed the clinical and ultrasound findings of 11 pathologically confirmed cases of cutaneous hidrocystoma. The aims of this study were to: (1) summarize the sonographic features of cutaneous hidrocystoma using conventional high-frequency ultrasound (5–15 MHz); (2) correlate imaging findings with histopathologic results; (3) explore the causes of preoperative misdiagnosis and key points of differential diagnosis; and (4) discuss the potential contribution of HFUS to lesion localization and preoperative surgical planning.

## 2 Materials and methods

### 2.1 Patients

Medical records of 13 patients with histopathologically confirmed cutaneous hidrocystoma who had undergone preoperative ultrasound examination at our institution between April 2022, and December 2023 were reviewed. Two patients were excluded: one due to coexistence of basal cell carcinoma in the lesion area, and one due to incomplete ultrasound imaging data. Finally, 11 patients were included. The detailed demographic and clinical characteristics of all patients are summarized in Table 1.

**Table 1:** Demographics and clinical characteristics of the patients.

Patient no	Age	Sex	Lesion location	Lesion size (mm)	Clinical presentation	Duration	Ultrasound interpretation	Final pathology
1	27	M	Urethral meatus	16×10×14	Mass at the urethral meatus present for 20 + years, gradually enlarging with tenderness	20 + Years	Hemangioma	Hidrocystoma
2	46	M	Scalp	12×9×5	Scalp nodule noted for over 2 years	2 + Years	Subcutaneous nodule	Hidrocystoma
3	47	F	Left facial area	7×7×5	Facial mass noted for over 3 years	3 + Years	Left facial nodule	Hidrocystoma
4	59	F	Right shoulder	10×6×10	Nodule on the right shoulder present for several years	Several years	Epidermoid cyst	Hidrocystoma
5	54	F	Lateral aspect of right eye	7×4×6	Subcutaneous nodule on the lateral aspect of right eye present for 4 + years, slowly growing, asymptomatic	4 + Years	Hemangioma	Hidrocystoma
6	36	M	Left cheek	7×7×7	Left cheek mass noted for 2 days	2 Days	Subcutaneous cyst	Hidrocystoma
7	62	F	Mandible	6×3×5	Subcutaneous mass on the mandible present for 5 years	5 Years	Subcutaneous cystic lesion	Hidrocystoma
8	49	M	Head	8×4×8	Head subcutaneous mass noted for over 10 years	10 + Years	Subcutaneous nodule on the head, most likely benign	Hidrocystoma
9	71	M	Head	11×4×10	Head subcutaneous mass noted for over 10 years	10 + Years	Epidermoid cyst	Hidrocystoma
10	47	F	Right back	7×4×7	Right back subcutaneous nodule noted for over 2 years	2 + Years	Epidermoid cyst	Hidrocystoma
11	30	M	Right frontal area	13×12×5	Forehead mass noted for over 1 year	1 + Years	Epidermoid cyst	Hidrocystoma

The cohort consisted of 6 men and 5 women, with an age range of 27–71 years (mean, 48.0 ± 12.3 years). Most patients presented with incidentally discovered, painless superficial nodules that were cystic on palpation and demonstrated slow growth. Lesions were most commonly located in the head and neck region (8/11, 72.7%), including the eyelid (*n* = 3), cheek (*n* = 4), and forehead (*n* = 1). The remaining lesions were located in the back, abdomen, and external urethral orifice (one case each).

### 2.2 Clinical examination

All patients underwent initial clinical examination by board-certified dermatologists. Clinical characteristics including lesion location, size, color, surface features, and palpation findings were documented. Dermoscopy was not routinely performed as part of this retrospective analysis, as the primary focus was on ultrasound characterization.

### 2.3 Ultrasound examination

All ultrasound examinations were performed in the Department of Radiology by two board-certified radiologists with more than 10 years of experience in superficial soft-tissue imaging.

High-frequency color Doppler ultrasound was performed using the following commercially available systems and transducer specifications:

GE LOGIQ E9: ML6-15 linear-array transducer (6–15 MHz).

GE LOGIQ E11: ML6-15 linear-array transducer (6–15 MHz).

Mindray Resona 7EXP: L14-5WU linear-array transducer (5–14 MHz).

During examination, patients were positioned comfortably with full exposure of the lesion site. A thick layer of ultrasound coupling gel was applied to ensure adequate acoustic coupling without compression of the superficial lesion. The lesions were evaluated using multiplanar, continuous, and dynamic scanning with minimal probe pressure to avoid compression artifacts.

Preset “superficial lesion” settings were used, with adjustments to gain, depth, and focal zones to optimize image quality and contrast resolution. The focal zone was positioned at the level of the lesion to maximize lateral resolution.

The following sonographic features were systematically recorded:

- *Morphologic characteristics*: Lesion location, size (measured in three orthogonal dimensions: length, width, and depth), shape (regular or irregular), margin (well-defined or ill-defined), and relationship to the dermis and subcutaneous tissue.
- *Internal echogenicity*: Echogenic pattern (anechoic, hypoechoic, isoechoic, or mixed echogenicity), as well as the presence of septations, mural nodules, calcifications, and posterior acoustic features (enhancement or attenuation).
- *Vascularity*: Color Doppler flow imaging (CDFI) was used to assess the presence, distribution (peripheral or internal), and degree of vascularity.

Vascularity was graded using a 4-point scale: Grade 0 (avascular - no detectable flow), Grade I (minimal - < =3 discrete flow signals, predominantly peripheral), Grade II (moderate - multiple flow signals or continuous flow in <50% of lesion), and Grade III (marked - continuous flow in > = 50% of lesion or circumferential encasing pattern). For lesions with detectable blood flow, spectral Doppler analysis was performed to measure flow velocity and resistance index (RI).

In cases of disagreement between the two radiologists, a consensus was reached through joint image review and discussion. All ultrasound images were archived for subsequent analysis.

### 2.4 Histopathologic examination

All surgical specimens were processed according to standard histopathologic protocols. Tissues were fixed in 10% neutral buffered formalin, embedded in paraffin, sectioned at 4-microm thickness, and stained with hematoxylin and eosin (H&E). Histopathologic evaluation was performed by board-certified pathologists who were blinded to the ultrasound findings.

The histopathologic criteria for identifying hidrocystoma origin included the following features: cyst wall composition (single-layered or multilayered epithelium), epithelial lining type with cytologic features indicating eccrine or apocrine differentiation (eccrine hidrocystoma: bilayered lining with basally located round nuclei and moderate pale cytoplasm, absence of decapitation secretions or apical snouts; apocrine hidrocystoma: a dual layer composed of outer myoepithelial cells and inner columnar cells with decapitation secretions and apical snouting), cyst contents (serous fluid, mucinous material, cellular debris, hemorrhage), presence of papillary projections, wall thickness, and any secondary changes (inflammation, fibrosis). Immunohistochemical staining for CK7, CK18, GCDFP-15, S100, and PAS was performed when necessary to confirm differentiation type.

### 2.5 Interpretation of ultrasound findings

Given the case-series design and the absence of established ultrasound diagnostic criteria for cutaneous hidrocystoma, preoperative ultrasound interpretations were summarized descriptively and compared with postoperative histopathologic diagnoses. We recorded whether each lesion had been interpreted preoperatively as hidrocystoma, as a nonspecific cystic lesion, or as another type of superficial lesion. No formal diagnostic-performance analysis was performed. This descriptive approach was used because cutaneous hidrocystoma is rare and its ultrasound features have been reported mainly in limited case-based studies (6).

## 3 Results

A total of 11 lesions were identified in 11 patients. The individual patient characteristics and ultrasound interpretations are detailed in Table 1, while the aggregated sonographic features are presented in Table 2.

**Table 2:** Distribution of the hidrocystomas with different sonographic features.

Feature category	Finding	*n* (%)	Notes
Location	Dermis only	11 (100%)	All superficial
	Subcutaneous extension	3 (27.3%)	
Size	<10 mm	7 (63.6%)	
	10–15 mm	3 (27.3%)	
	>15 mm	1 (9.1%)	Single outlier
Shape	Round/hemispherical	9 (81.8%)	
	Irregular	2 (18.2%)	
Margins	Well-defined	8 (72.7%)	
	Ill-defined	3 (27.3%)	
Internal echogenicity	Anechoic (simple cyst)	3 (27.3%)	With posterior enhancement
	Homogeneously hypoechoic	2 (18.2%)	
	Heterogeneous/mixed	4 (36.4%)	Septations/debris present
	Hypoechoic with papillary projections	2 (18.2%)	
Vascularity (CDFI)	Avascular (Grade 0)	9 (81.8%)	
	Minimal flow (Grade I)	1 (9.1%)	Indeterminate significance
	Prominent peripheral (Grade III)	1 (9.1%)	Circumferential “encasing” pattern
Posterior features	Acoustic enhancement	3 (27.3%)	Simple cysts only
	No enhancement	8 (72.7%)	

### 3.1 General sonographic features

**Figure 1:**
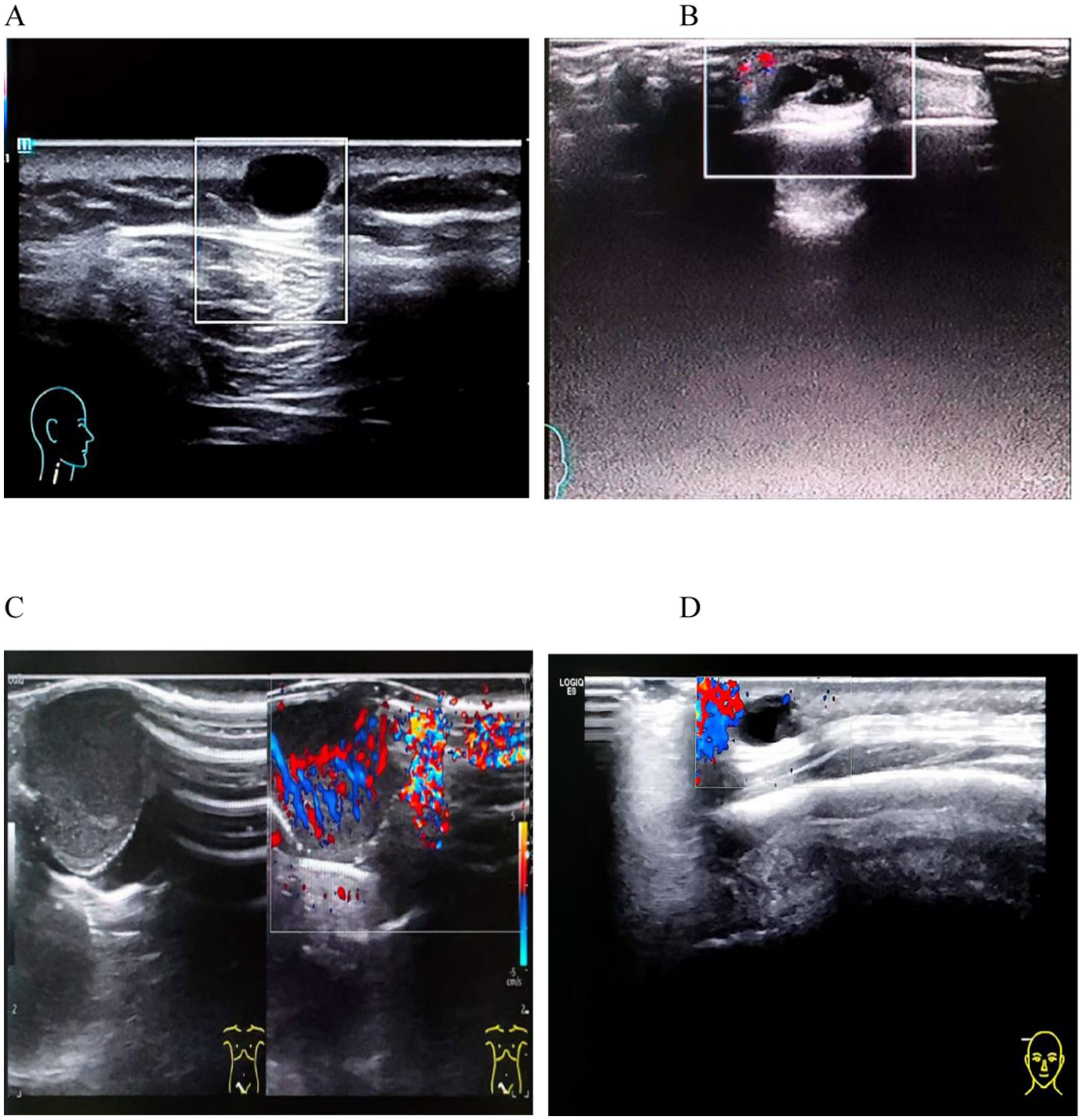
Representative high-frequency ultrasound images of cutaneous hidrocystoma. Panel A: Grayscale neck soft tissue ultrasound demonstrating a well-circumscribed hypoechoic lesion framed by a white box. Panel B: Corresponding Doppler ultrasound revealing focal vascular flow adjacent to the lesion. Panel C: Split-view ultrasound of the external urethral meatus, with grayscale on the left and color Doppler on the right, showing extensive intralesional vascular signals. Panel D: Longitudinal grayscale head ultrasound with focal Doppler vascular signals beside the hypoechoic lesion.

High-frequency ultrasound clearly demonstrated that all lesions were located within the dermis. The comprehensive sonographic feature analysis is presented in Table 2. Most lesions measured less than 1.5 cm in maximum diameter (10/11, 90.9%), including 7 lesions (63.6%) measuring less than 1.0 cm. The lesions were predominantly round or hemispherical in shape (9/11, 81.8%), with well-defined margins observed in 8 cases (72.7%). Representative ultrasound images illustrating these features are presented in Figure 1.

Internal echogenicity varied among lesions. Three lesions (27.3%) exhibited typical cystic features, appearing anechoic with posterior acoustic enhancement (Figure 1A). One lesion (9.1%) was homogeneously hypoechoic, and another (9.1%) was mildly hypoechoic. Four lesions (36.4%) demonstrated heterogeneous echogenicity, with internal fine punctate echoes, septations, and papillary projections (Figure 1B). These findings suggest relatively thick internal contents or possible secondary changes such as hemorrhage or infection. Two lesions (18.2%) showed hypoechoic appearance with papillary projections (Figure 1D).

### 3.2 Vascular characteristics

Color Doppler flow imaging (CDFI) showed no detectable internal or peripheral vascular signals in the majority of lesions (9/11, 81.8%). The vascularity grading distribution is detailed in Table 2, with the grading system defined in Table 3. Vascular signals were identified in 2 lesions (18.2%). One lesion demonstrated minimal vascularity and was considered indeterminate (Grade I; 9.1%). The other lesion exhibited prominent peripheral vascularity with a characteristic circumferential “encasing” distribution (Grade III; 9.1%) (Figure 1C). Spectral Doppler analysis of this lesion revealed a low-resistance arterial waveform with a resistance index (RI) of 0.45–0.55, along with a continuous venous flow pattern.

**Table 3:** Vascularity grading system definition.

Grade	Description	Criteria	*n* in this study
0 (Avascular)	No detectable flow	No color Doppler signals in lesion or periphery	9
I (Minimal)	Focal/spotty flow	<=3 discrete flow signals, predominantly peripheral	1
II (Moderate)	Moderate flow	Multiple flow signals or continuous flow in <50% of lesion	0
III (Marked)	Abundant flow	Continuous flow in > = 50% of lesion or circumferential encasing pattern	1

### 3.3 Correlation between sonographic and histopathologic findings

Histopathologic examination confirmed hidrocystoma in all 11 cases. Detailed histopathologic features and their correlations with sonographic findings are presented in Table 4. The histopathologic subtypes were apocrine hidrocystoma (*n* = 7, 63.6%) and eccrine hidrocystoma (*n* = 4, 36.4%). Cyst wall composition was multilayered epithelium in 9 cases (81.8%) and single-layered in 2 cases (18.2%). Cyst contents included serous fluid (*n* = 3, 27.3%), mucinous material (*n* = 4, 36.4%), cellular debris with hemorrhage (*n* = 3, 27.3%), and mixed contents (*n* = 1, 9.1%). Papillary projections from the cyst wall were observed in 4 cases (36.4%).

**Table 4:** Correlation between sonographic and histopathologic findings.

Sonographic finding	Histopathologic correlate	Cases (*n*)
Anechoic with posterior enhancement	Simple cyst with clear serous fluid	3
Homogeneously hypoechoic	Cyst with homogeneous proteinaceous content	2
Heterogeneous with fine punctate echoes	Cyst with cellular debris/hemorrhage	2
Internal septations	Papillary projections from cyst wall	2
Peripheral “encasing” vascularity	Proliferative cyst wall with vascularized stroma	1
Mural nodules	Papillary epithelial proliferation	2

**Figure 2:**
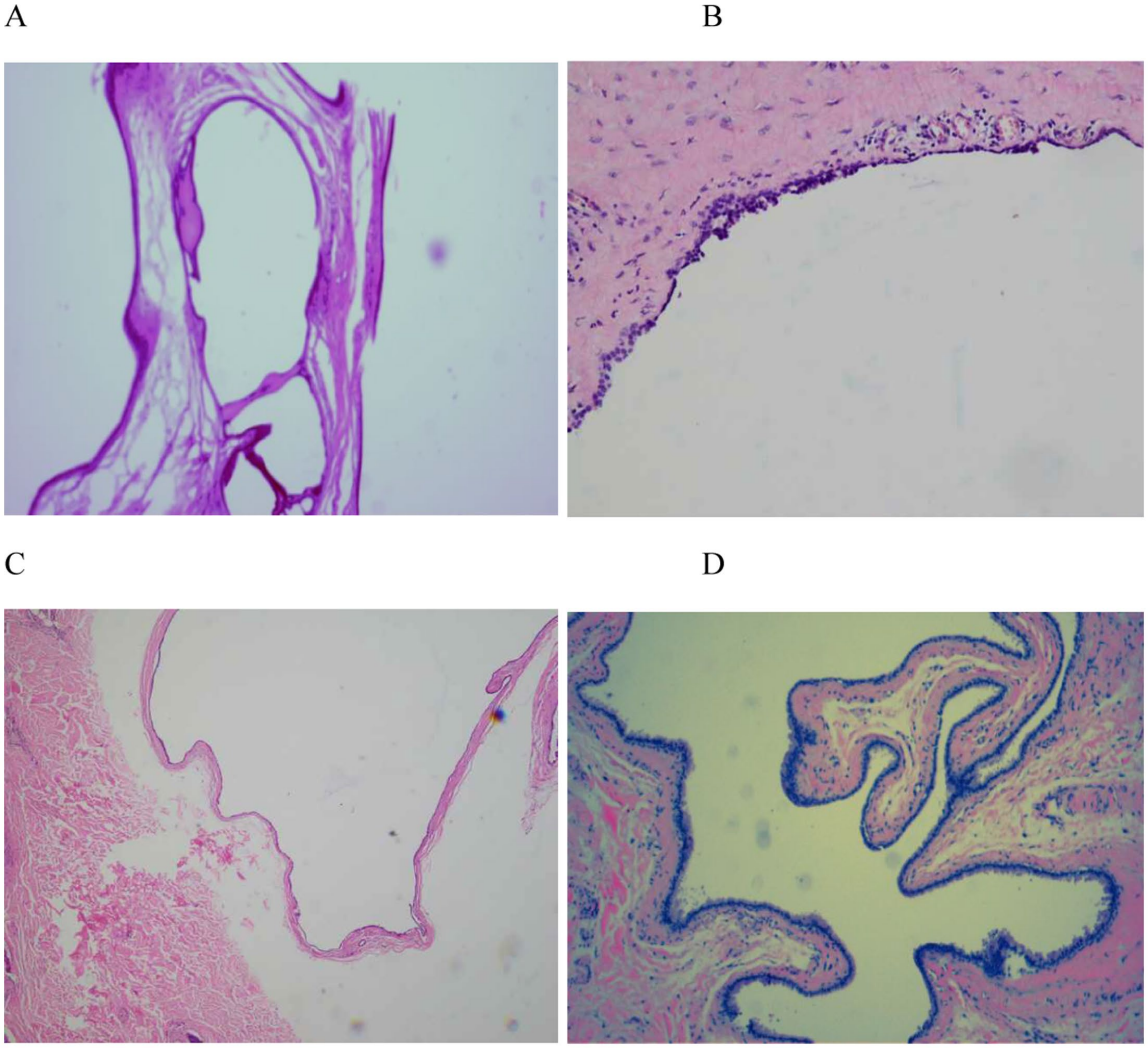
Representative histopathologic images of cutaneous hidrocystoma (hematoxylin and eosin staining, ×100 magnification). **(A)** Apocrine hidrocystoma (Patient 2, scalp) showing a cyst lined by dual layer composed of outer myoepithelial cells and inner columnar cells with decapitation secretions and apical snouting. Intraluminal homogeneous eosinophilic secretions are present. **(B)** Eccrine hidrocystoma (Patient 1, urethral meatus) demonstrating cyst lined by bland bilayer with basally located round nuclei and moderate pale cytoplasm, absence of decapitation secretions. The cyst contains serous fluid with cellular debris. **(C)** Apocrine hidrocystoma with papillary projections (Patient 10, back) showing cyst wall with papillary epithelial proliferation projecting into the lumen. The cyst contains mucinous material and cellular debris. **(D)** Apocrine hidrocystoma with proliferative changes (Patient 5, lateral aspect of right eye) demonstrating multilayered epithelial lining with vascularized stroma in the cyst wall, corresponding to the peripheral “encasing” vascularity observed on ultrasound.

The sonographic-histopathologic correlations were as follows: anechoic cysts with posterior enhancement corresponded to simple cysts with clear serous fluid (3 cases); heterogeneous echogenicity with internal echoes and septations correlated with cysts containing cellular debris, hemorrhage, or mucinous material (4 cases); hypoechoic lesions with papillary projections corresponded to cysts with papillary epithelial proliferation from the cyst wall (2 cases); and the lesion with peripheral “encasing” vascularity showed proliferative cyst wall with vascularized stroma on histopathology (1 case), as detailed in Table 4. Representative histopathologic images are presented in Figure 2.

### 3.4 Preoperative ultrasound interpretation

Preoperative ultrasound interpretations included nonspecific cystic lesion in 3 cases (27.3%), epidermoid cyst in 4 cases (36.4%), hemangioma in 2 cases (18.2%), and hypoechoic solid nodule in 2 cases (18.2%). Table 5 summarizes these preoperative ultrasound interpretations. None of the lesions was interpreted as hidrocystoma before surgery. Although 3 lesions were described as cystic lesions, the specific diagnosis of hidrocystoma was not suggested preoperatively. HFUS nevertheless provided descriptive information regarding lesion depth, size, morphology, margin definition, vascularity, and relationship to adjacent structures.

**Table 5:** Correlation between ultrasound interpretation and pathological result.

Ultrasound interpretation	*n*	Pathology correlation	Comment
Cystic lesion (non-specific)	3	Hidrocystoma	Cystic nature was described, but hidrocystoma was not suggested
Epidermoid cyst	4	Hidrocystoma	Interpreted as another superficial cystic lesion
Hemangioma	2	Hidrocystoma	Likely related to vascular appearance
Hypoechoic solid nodule	2	Hidrocystoma	Nonspecific solid-appearing interpretation
Specific interpretation of hidrocystoma	0/11 (0%)		No lesion was interpreted as hidrocystoma before surgery

## 4 Discussion

### 4.1 Principal findings

This case series of 11 pathologically confirmed cutaneous hidrocystomas characterized the sonographic features using conventional high-frequency ultrasound (5–15 MHz). The principal findings were: (1) all lesions were located within the dermis; (2) sonographic appearances were highly variable, with heterogeneous echogenicity being the most common pattern (36.4%); (3) most lesions were avascular (81.8%), but peripheral vascularity with circumferential encasing pattern was observed in one case; (4) no lesion was correctly diagnosed as hidrocystoma preoperatively based on ultrasound alone; and (5) ultrasound provided valuable anatomic information for surgical planning in all cases.

### 4.2 Comparison with previous studies

The sonographic features observed in this study are consistent with previous reports of hidrocystoma, while extending the characterization beyond periocular lesions. Chin et al. (2003) (12) and Furuta et al. (2007) (13) described anechoic, well-defined, avascular hidrocystomas in the periocular region using ultrasound biomicroscopy (35–50 MHz). Our findings using conventional HFUS (5–15 MHz) show greater heterogeneity, which may reflect the wider anatomic distribution (head, neck, back, abdomen, genital area) and the lower frequency range with consequently different resolution characteristics.

Cinotti et al. (2024) (6) recently reported UHFUS (50 MHz) findings in hidrocystoma, describing anechoic or hypoechoic oval structures with thin hyperechoic borders and internal septa. Our study using lower frequencies showed similar basic morphology but greater internal echogenicity variability, likely due to differences in resolution and the inclusion of lesions with secondary changes (hemorrhage, debris) that may not be as apparent at higher frequencies.

### 4.3 Pathologic basis of sonographic heterogeneity

The marked variability in internal echogenicity observed in this cohort (anechoic 27.3%, hypoechoic 18.2%, heterogeneous 36.4%, papillary projections 18.2%) can be explained by histopathologic correlates. Anechoic cysts with posterior enhancement corresponded to simple cysts with clear serous fluid. Heterogeneous echogenicity with internal echoes and septations correlated with cysts containing cellular debris, hemorrhage, or mucinous material. Hypoechoic lesions with papillary projections corresponded to cysts with papillary epithelial proliferation from the cyst wall. These findings are consistent with the known histopathologic spectrum of hidrocystoma, which includes simple retention cysts, proliferative variants, and lesions with secondary changes (1, 3), as systematically correlated in Table 4.

The single case with prominent peripheral vascularity (Grade III, circumferential encasing pattern, RI 0.45–0.55) is particularly noteworthy. Histopathologic examination revealed proliferative cyst wall with vascularized stroma, suggesting that increased vascularity may occur in association with inflammatory changes or reactive proliferation. This finding explains the potential for misdiagnosis as hemangioma, which occurred in 2 of our 11 cases (18.2%), as quantified in Table 5.

### 4.4 Differential diagnosis considerations

Because cutaneous hidrocystoma is relatively uncommon and demonstrates variable sonographic appearances, it is frequently misdiagnosed preoperatively as other superficial lesions. Table 6 provides a structured comparison of key imaging features distinguishing hidrocystoma from its primary differential diagnoses: epidermoid cysts, hemangiomas, apocrine cystadenomas, sebaceous duct cysts, pilar cysts, folliculitis, and eccrine spiradenomas with secondary changes. The value of HFUS lies not only in lesion characterization but also in establishing this structured differential diagnostic framework through systematic comparison of imaging features, thereby narrowing the diagnostic spectrum and reducing misdiagnosis.

**Table 6:** Differential diagnosis among hidrocystoma and some common lesions.

Feature	Hidrocystoma	Epidermoid cyst	Hemangioma	Apocrine cystadenoma	Sebaceous duct Cyst	Pilar cyst	Folliculitis	Eccrine spiradenoma
Typical location	Dermis (head/neck)	Dermis/subcutis, often with epidermal connection	Any site, commonly head/neck	Axilla, groin, perianal (apocrine-rich regions)	Trunk, neck, proximal extremities	Scalp, face (most common)	Any hair-bearing area	Head, neck, trunk
Size	3–15 mm	Variable, often 5–30 mm	Variable, can be large	Often >15 mm	Variable, 5–30 mm	Variable, 5–30 mm	Variable	Variable
Shape	Round/hemispherical	Round/oval, often with “onion-skin” layering	Lobulated	Multiloculated, sometimes with papillary projections	Round/oval, well-defined	Round/oval, well-defined	Irregular, ill-defined	Round/oval, well-defined
Internal echogenicity	Variable: anechoic to heterogeneous with debris	Heterogeneous, “onion-skin” or lamellated	Reticular/honeycomb, compressible	Multiloculated, anechoic with internal echoes and solid components	Hypoechoic or anechoic, sometimes with internal echogenic foci	Homogeneous hypoechoic (homogeneous)	Central hypoechoic with peripheral changes	Hypoechoic, may have cystic degeneration
Vascularity	Usually avascular (82%); peripheral “encasing” if present (18%)	Usually avascular	Abundant internal flow, prominent feeding arteries	Variable, may be increased	Avascular	Avascular	Increased peripheral vascularity	Hypervascular (stromal vascularization)
Compressibility	Non-compressible	Non-compressible	Compressible	Non-compressible	Non-compressible	Non-compressible	Non-compressible	Non-compressible
Posterior features	Variable enhancement	Variable	Enhancement	Variable	Variable	No enhancement	Variable enhancement	Possible enhancement
Key distinguishing feature	Location + variable echogenicity	“Onion-skin” sign, epidermal connection	Compressibility + internal vascularity	Size, location, multiloculation, papillary projections	Clinical appearance, wavy wall, sebaceous material	Location on scalp, firm consistency, no bluish appearance	Inflammatory signs, central hair follicle	Pain + vascular pattern + histology

First, differentiation from epidermoid cysts is essential, as both may present as dermal cystic lesions. Epidermoid cysts are typically connected to the epidermis and often exhibit more complex internal echogenicity, including the characteristic “onion-skin” layered appearance or the “submarine sign” (focal projection of hypoechoic portion to epidermis) (5). In contrast, hidrocystomas arise from sweat glands, are relatively independent from the epidermis, and lack such layered structures. Both entities generally demonstrate minimal or absent vascularity on Doppler imaging (5, 13).

Second, differentiation from cutaneous hemangiomas represents the most challenging scenario, particularly when hidrocystomas exhibit peripheral “encasing” vascularity. Hemangiomas, which are more common in infants and children, typically present as lobulated lesions with internal reticular or honeycomb-like architecture. They usually demonstrate abundant internal vascularity, often with prominent feeding arteries, and show high-velocity, low-resistance arterial waveforms on spectral Doppler imaging. Additionally, hemangiomas are compressible under probe pressure. These features differ fundamentally from hidrocystomas, which--even when vascularized--tend to show peripheral rather than internal flow and maintain a relatively regular morphology (11, 14, 15).

Third, differentiation from apocrine cystadenomas is important given their shared apocrine origin and cystic nature. Apocrine cystadenomas typically present as larger, multiloculated cystic lesions, often with more prominent papillary projections and occasional solid components. They may demonstrate increased vascularity compared to simple hidrocystomas and are more commonly located in the axilla, groin, or perianal regions, where apocrine glands are concentrated (1, 3).

Fourth, sebaceous duct cysts (steatocystomas) should be considered in the differential diagnosis. These lesions arise from sebaceous duct epithelium and typically present as dermal or subcutaneous cysts with thin, wavy walls. On ultrasound, they may appear as well-defined hypoechoic or anechoic lesions, sometimes with internal echogenic foci representing sebaceous material. They are usually avascular and most commonly occur on the trunk, neck, or proximal extremities (1, 4).

Fifth, pilar cysts (trichilemmal cysts) are derived from the outer root sheath of the hair follicle and typically present as firm, mobile, well-circumscribed nodules, most commonly on the scalp. On ultrasound, they appear as well-defined hypoechoic lesions within the dermis or superficial subcutaneous tissue, often with homogeneous internal echogenicity and no vascularity. Unlike hidrocystomas, they lack the translucent bluish clinical appearance and are not associated with sweat gland origin (1, 4).

Sixth, folliculitis with abscess formation may mimic hidrocystoma, particularly when the latter presents with secondary inflammatory changes. Folliculitis typically shows a central hair follicle with surrounding hypoechoic inflammatory changes, often with increased peripheral vascularity on Doppler imaging. Clinical history of inflammation, tenderness, and erythema helps distinguish these entities (1, 4).

Seventh, eccrine spiradenomas with secondary cystic degeneration may present diagnostic challenges. These lesions typically appear as well-defined hypoechoic dermal nodules with possible posterior acoustic enhancement. They often demonstrate hypervascularity on Doppler imaging due to their highly vascularized stroma, and may be painful on palpation. Histologically, they show lobular proliferation of basaloid cells with two distinct cell types (dark peripheral cells and pale central cells), which distinguishes them from hidrocystomas (1, 4, 16).

### 4.5 Clinical implications

Although HFUS did not allow specific preoperative identification of cutaneous hidrocystoma in this case series, it provided useful anatomic information regarding lesion depth, size, internal architecture, vascularity, and relationship to adjacent structures. Such information may assist preoperative assessment, particularly when lesions are located in cosmetically or anatomically sensitive areas, such as the periocular or facial region. Therefore, the role of HFUS in this setting should be understood primarily as lesion characterization and preoperative localization rather than definitive diagnosis.

### 4.6 Limitations

This study has several limitations that should be acknowledged. First, the sample size was small (*n* = 11), which is inherent to the rarity of this condition but limits generalizability. Second, the study was retrospective, which may introduce selection bias toward surgically managed cases. Third, subgroup analysis by histologic subtype (eccrine vs. apocrine) was not performed due to limited numbers, though this represents an important area for future investigation. Fourth, the ultrasound frequencies used (5–15 MHz) are lower than those employed in dedicated dermatologic ultrasound (> = 15 MHz), which may limit resolution of fine architectural details. Finally, no dermoscopy or other adjunctive clinical imaging was routinely performed, precluding direct comparison of sonographic findings with other adjunctive imaging modalities.

## 5 Conclusion

This case series demonstrates that cutaneous hidrocystomas show variable sonographic appearances on conventional high-frequency ultrasound. Although none of the lesions was specifically interpreted as hidrocystoma before surgery, HFUS provided useful information regarding lesion location, depth, internal architecture, and vascularity. These findings may help narrow the differential diagnosis and support preoperative planning. Larger prospective studies using dedicated dermatologic ultrasound and multimodal assessment are needed to further define the sonographic spectrum of cutaneous hidrocystoma.

## Data Availability

The raw data supporting the conclusions of this article will be made available by the authors, without undue reservation.
